# Temporal and spatial isotopic variability of marine prey species in south-eastern Australia: Potential implications for predator diet studies

**DOI:** 10.1371/journal.pone.0259961

**Published:** 2021-11-30

**Authors:** Marlenne A. Rodríguez-Malagón, Cassie N. Speakman, Grace J. Sutton, Lauren P. Angel, John P. Y. Arnould

**Affiliations:** School of Life and Environmental Sciences, Deakin University, Burwood, Victoria, Australia; MARE – Marine and Environmental Sciences Centre, PORTUGAL

## Abstract

Stable isotope analyses, particularly of carbon (*δ*^13^C) and nitrogen (*δ*^15^N), are used to investigate ecological relationships among species. For marine predators, research has shown the main factors influencing their intra-specific and intra-individual isotopic variation are geographical movements and changes in the composition of diet over time. However, as the differences seen may be the result of changes in the prey items consumed, a change in feeding location or the combination of both, knowledge of the temporal and spatial consistency in the isotopic values of prey becomes crucial for making accurate inferences about predator diets. This study used an abundant marine predator, the Australasian gannet (*Morus serrator*), as prey sampler to investigate the annual variation in fish and squid prey isotope values over a four-year period (2012–2015) and the geographic variation between two sites with contrasting oceanographic conditions. Significant inter-annual variation was observed in *δ*^13^C and/or *δ*^15^N values of five of the eight prey species analysed. The strongest inter-annual variation in both *δ*^13^C and *δ*^15^N values occurred in 2015, which coincided with a strong El Niño-Southern Oscillation (ENSO). This may suggest a temporal fluctuation in the geographic source of prey or the origin of their nutrients. These results suggest that it is important to consider the potential significant differences in isotopic values within the prey assemblages that predators consume. This is important to improve the interpretation of marine predator isotope results when determining the influence of environmental variability on their diets.

## Introduction

Stable isotope analyses are powerful tools for understanding the trophic niche of animals and are widely used in ecology, providing important information for conservation [1]. In particular, the stable isotope ratios of carbon (^13^C/^12^C) and nitrogen (^15^N/^14^N) are commonly used to describe the trophic niche of species [2], to reconstruct animal diets [3, 4], and to make inferences about foraging area and temporal diet variations [5, 6]. This is possible due to the variation in *δ*^13^C values, for example between plant species with different photosynthetic pathways (i.e. C_3_ or C_4_ plants [7]) or differences in isotopic baselines between nearshore vs offshore and benthic vs pelagic in the marine environment [8, 9], that serve to determine the location of primary sources of food. Similarly, the ^15^N enrichment of consumers relative to their food items serves as an indicator of the consumer’s trophic position [10]. These differences are believed to be due to the fractionation of the heavier isotope (^15^N) from the isotopically lighter isotope (^14^N) during amino acid synthesis, resulting in the retention of the heavier isotope and the excretion of the lighter [11].

In recent years, the use of isotopic analysis to study predator diets has increased in response to improved statistical tools such as mixing models (which consider the isotopic composition of consumers and their foods) to make inferences about the composition of the animal’s assimilated diet [12] and knowledge associated with isotopic enrichment processes [10]. Such research has shown that the main factors influencing intra-specific and intra-individual variation in stable isotopic values are the geographical movement of predators [13, 14] and/or changes in the composition of their diet over time [15, 16]. However, without knowledge of the isotopic signatures of potential prey and how these vary spatially and temporally, interpreting intra-specific and intra-individual differences in predator isotopic values is problematic [17, 18]. For instance, isotopic differences seen in predators may be the result of changes in the prey items consumed, a change in feeding location, or a combination of both. Furthermore, prey isotopic values within the same location may change over time if the prey items on which they depend also varies as a consequence of biogeochemical processes [19, 20]. Hence, knowledge of the temporal and spatial consistency in the isotopic values of prey is crucial for making accurate inferences about predator diets [21, 22].

Within marine environments, top predators play an important role as top-down controllers of prey species, nutrient cyclers and ecosystem engineers [23]. Marine environments are complex and dynamic and their temporal and spatial variation influences the ecology of marine life [24]. At local and regional scales, physical features such as water currents, bathymetry, tide regimes and nutrient fluxes determine the structure of marine and coastal ecosystems and influence the behaviour and distribution of marine fauna [25]. Concurrently, naturally occurring stable isotopes are influenced by water temperature and phytoplankton photosynthetic pathways [13, 26], and by N_2_ fixation processes in the ocean surface, terrestrial runoff and atmospheric precipitation [27, 28] for *δ*^13^C and *δ*^15^N, respectively. These factors influence the isotopic values of marine plankton that could potentially produce spatial isotopic variation at the base of marine food chains [29]. In addition, particularly for *δ*^13^C values, local temporal isotopic variation has also been reported in relation to changes in currents and nutrient availability influencing primary productivity of marine food chains [22, 29, 30]. Consequently, knowledge of the temporal and spatial variation in prey isotopic values is especially important when examining marine predator diets.

In south-eastern Australia, Bass Strait is a key area of marine predator biodiversity and one of the fastest warming regions on eath. It is influenced by 3 main ocean currents, namely the warm East Australian Current (EAC) and South Australian Current (SAC) which mix with the subantarctic surface water (SASW) [31]. Previous studies have documented significant inter-annual and geographic differences in the stable isotope values of marine predators within Bass Strait (e.g. Australian fur seals *Arctocephalus pusillus doriferus*, little penguins *Eudyptula minor* and Australasian gannets *Morus serrator*) suggesting variation in their diets in relation to temporal and spatial factors [32–35]. However, isotopic information on the many potential prey species in Bass Strait is limited [36, 37]. In addition, there is currently no information on the temporal or spatial variation in the region’s baseline values for isotopes [13, 38]. Consequently, it is not possible to ascertain whether variations in predator isotopic values reflect changes in diet species composition, foraging areas or a combination of these. Information about the isotopic variation of regional marine predators is crucial for understanding how current oceanic variability influences their diets and predicting how their populations may respond to future ecosystem changes.

Using an abundant marine predator, the Australasian gannet, as a prey collection agent, the primary objective of the present study was to investigate the temporal and spatial variation in *δ*^13^C and *δ*^15^N isotopic values in an assemblage of common gannet prey species found in south-eastern Australia. Specifically, the aims were to: 1) determine inter-annual variation in prey isotope values over a four year period (2012–2015); and 2) assess geographic variation between two sites with contrasting oceanographic conditions.

## Materials and methods

### Sample collection

**Fig 1:**
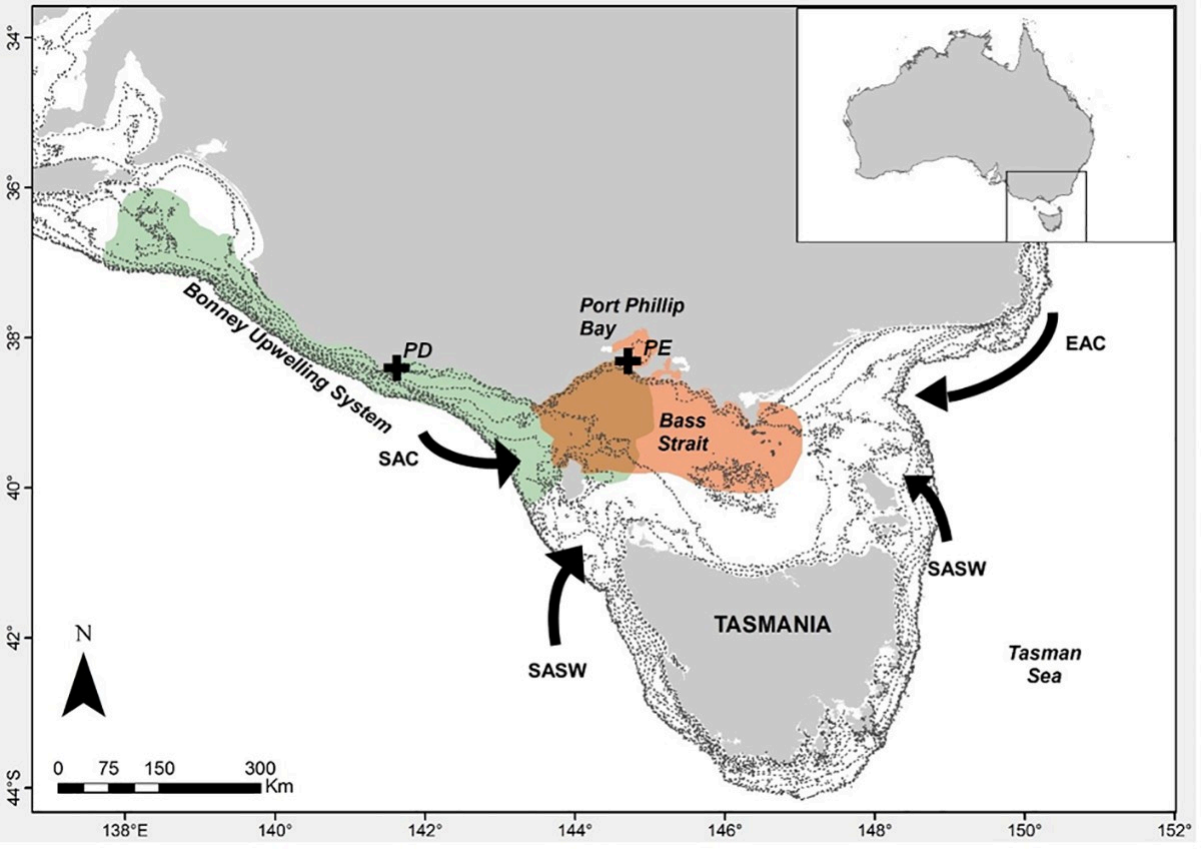
Location of the Point Danger (PD) and Popes Eye (PE) Australasian gannet breeding colonies (black crosses). The main water masses that influence Bass Strait are indicated by the arrows. The South Australian Current (SAC, winter) and East Australian Currents (EAC, winter and summer) bring warm and low nutrient waters into the marine region, while the Sub-Antarctic Surface Water (SASW, summer) drives cold and productive waters from the south. PD (green) and PE (orange) Australasian gannets (*Morus serrator*) foraging ranges obtained from GPS data [40]. Bathymetric contours (every 20 m) within the continental shelf are shown.

The Australasian gannet, a top marine predator, was used as a means of prey sample collection in northern Bass Strait. Samples were collected from individuals at the Point Danger (PD, 38° 23’ 36.09” S, 141° 38’ 55.94” E) and Pope’s Eye (PE, 38° 16’ 35.88” S, 144° 41’ 56.21” E) breeding colonies (Fig 1). During the breeding season, adult birds from PD have been shown to forage within the continental shelf, ranging up to 238 km north-west and south-east from the colony, hunting within limits of the Bonney Upwelling system [25, 32]. In contrast, birds from PE forage within the shallow (average depth < 13.6 m [39] waters of Port Phillip Bay, outside the bay within central Bass Strait, or in both habitats [32, 40]. The diet of Australasian gannets within Bass Strait has been reported to comprise at least 37 demersal/reef-associated and pelagic/oceanic species of fish and squid [41–45]. Hence, the location of these colonies, the foraging range of this predator and its broad diet allow for prey species representative of a wide range of habitats to be sampled.

As part of concurrent studies on the foraging ecology of Australasian gannets during the 2012–2015 breeding seasons (October-March) in each of three breeding stages: incubation; early chick-rearing (chick age 0–50 d); and late chick-rearing (chick age >50 d) [46], voluntary regurgitations by birds upon handling were collected in plastic bags and stored frozen (-20° C) until analysis in the laboratory. In the laboratory, regurgitate samples were thawed and prey specimens were identified to the lowest possible taxonomic level using published guides [47]. From complete prey only, standard length of individual specimens were recorded using Vernier callipers (± 0.1 mm) or metal ruler (± 1 mm) and body mass was measured on a top-loading balance (± 0.01 g, Ohaus Corporation, Parsippany USA). From incomplete samples, otoliths and squid beaks were extracted, where possible, to confirm fish and squid identification and to estimate their standard length using published regression equations [48, 49]. Depending on the size of the prey specimen, 1–4 g of muscle tissue was collected from all individual prey samples. For fish, tissue was sampled posterior to the anus, above the lateral line, on one side of the vertebral column. For squid, tissue was taken from the base of the mantle.

### Stable isotope analysis

All tissue samples were oven-dried at 50° C for at least 24 h or until the dry weight remained stable. Samples were then ground into powder using a mortar and pestle and lipids were extracted by adding a 2:1 chloroform–methanol mixture to the powdered samples. Samples were then shaken using a vortex mixer and centrifuged for 10 min at 10°C (2500 rpm). The supernatant was discarded and the procedure repeated at least once or until the supernatant was clear and colourless after centrifuging [50, 51]. Samples were then dried for 24 h under a fume hood. Once dry, they were further refined into a powder and 1.0 mg of each sample was loaded into separate tin capsules. Stable isotope analysis of carbon and nitrogen were conducted at the Farquhar laboratory of the Research School of Biology, Australian National University (Canberra, Australia). Samples were combusted in a CHN elemental analyser (CE1110, Carlo Erba) and resulting gases were analysed using an interfaced isoprime continuous-flow isotope ratio mass spectrometer (Micromass instruments). Quality control samples were run before and after each sequence using laboratory standards of sucrose ANU (-10.45 ‰) and BEET (-24.62 ‰) for *δ*^13^C and amino acids Alanine, Glycine and Cysteine used for *δ*^15^N; which provided replicate measurement errors of ±0.1‰ and 0.3 ‰, respectively. Stable isotope values were expressed in *δ*-notation as the deviation from standards in parts per mil (‰) according to the following equation:

$$\delta X=\left[(\frac{R_{sample}}{R_{standard}})–1\right]$$ (1)

where, *X* is ^13^C or ^15^N and *R* is the corresponding ratio of ^13^C/^12^C or ^15^N/^14^N. *R*_standard_ values were based on international standards Vienna Pee Dee Belemnite (VPDB) for *δ*^13^C, and atmospheric nitrogen (N_2_) in air for *δ*^15^N. The mean C:N mass ratio for all samples was calculated to be 3.17 (± 0.15 SD), indicating lipid concentrations are uniformly low and no data normalization is needed [52].

### Statistical analysis

All data processing and statistical analysis were conducted in R version 3.4.1 [53]. To evaluate simultaneously the effect of years (from 2012 to 2015) and colonies (PD and PE) in the prey isotopic niches, two-way ANOVA tests with interaction terms were performed considering *δ*^13^C and *δ*^15^N as response variables independently for each species. If prey were not sampled at both colonies, a one-way ANOVA test to evaluate the effect of year was performed. The assumptions of homegeniety of variance and normality were checked by visualising residual vs fitted plots and quantile plots, respectively. As sample sizes varied considerably among years and colonies for each species, an unbalanced design with Type-III sums of squares was considered when running this statistical analysis. The function *Anova* of the *car* package version 2.1–5 was used for such purposes [54]. Significant terms were tested using a posteriori multiple comparison test with the *Tukey HSD* function of the *stats* package version 3.4.1 [53].

To investigate whether habitat (benthic *versus* pelagic) influenced variation in stable isotope values within species, the coefficient of variation of *δ*^13^C and *δ*^15^N absolute values was calculated as a proxy for intra-species variation. The coefficients of variation were estimated by year and colonies for species with three or more samples. The equality of the estimated coefficients of variation was tested using the *asymptotic_test2* function of the *cvequality* package for summary statistics [55]. Each prey species was classified by habitat according to its biological information available on the Fishes of Australia database (https://fishesofaustralia.net.au/).

## Results

A total of 288 individual birds belonging to PD and PE colonies (143 and 145, respectively) were captured as part of a foraging behaviour study over the 2012–2015 breeding seasons. From them, 404 regurgitated samples (207 from PD and 197 from PE) were obtained (containing from 1 to 4 prey species each), from which, 1,181 individual prey items were collected (704 from PD and 477 from PE). Of these, 427 prey items were sufficiently undigested for stable isotope analysis (Table 1).

**Table 1:** Sample sizes of all the prey species found shown by year and colony. An asterisk indicates those species whose isotopic values were statistically tested for temporal and spatial differences. Habitat (pelagic, P or benthic, B) and diet for each prey species is listed (source: [56]).

Species (Scientific name)	Habitat and diet	2012		2013		2014		2015		Total
		PD	PE	PD	PE	PD	PE	PD	PE	
Australian anchovy (*Engraulis australis*)*^,c^	P, Zooplankton					1	8	19	15	43
Australian sardine (*Sardinops sagax*)*^,c^	P, Zooplankton and phytoplankton	1			2	32	21	12	19	87
Barracouta (*Thyrsites atun*)*^,a^	P, Cephalopods, pelagic fish and invertebrates	3	1		5	21	16	12	18	76
Blue mackerel (*Scomber australasicus)*^b^	P, Small fish and squid, pelagic invertebrates				1		7		2	10
Blue sprat (*Spratelloides robustus)*^b^	P, Zooplankton					3				3
Blue weed-whiting (*Haletta semifasciata*)^b^	B, Invertebrates and plant matter				1				1	2
Bluespotted goatfish (Upeneichthys vlamingii)*^,b^	B, Invertebrates and small fish		2		8		15		10	35
Eastern Australian salmon (*Arripis trutta)*^a^	P, Fish		2				1			3
Flathead (*Platycephalus* sp.*)*^b^	B, Fish and crustaceans				2				1	3
Gould’s squid (Nototodarus gouldi)*^,a^	P, Fish, crustaceans and cephalopods	2			1	10	2	15		30
Jack mackerel (Trachurus declivis)*^,.b^	P, Zooplankton, crustaceans and invertebrates		8		2	7	21	3	4	45
King gar (Scomberesox saurus)*^,a^	P, Zooplankton and fish larvae	6				12		9		27
Longsnout boarfish (*Pentaceropsis recurvirostris)*^b^	B, Polychaete worms, sea stars and algae					1				1
Redbait (Emmelichthys nitidus)*^,b^	P, Zooplankton, crustaceans and invertebrates	2				29		20		51
Snook (*Sphyraena novaehollandiae)*^a^	P, Small fish and invertebrates						1			1
Southern garfish (*Hyporhamphus melanochir)*^a^	P, Invertebrates and plant matter						5		1	6
Velvet leatherjacket (*Meuschenia scaber)*^c^	B, Invertebrates and plant matter					3				3
Yellowfin goby (*Acanthogobius flavimanus*)^a^	B, Crustacean and fish								1	1
Total		14	13	0	22	119	97	90	72	427

^a^ Migratory/highly mobile species.

^b^ non-migratory species.

^c^ age differences in habitat use: young = inshore, adults = open sea.

A total of 18 prey species were identified. Eight prey species were exclusively collected from PE, while five species were exclusively collected from PD. The means (± SE) of the standard length and body mass for each species in each year and colony can be found in the S1 Table.

**Fig 2:**
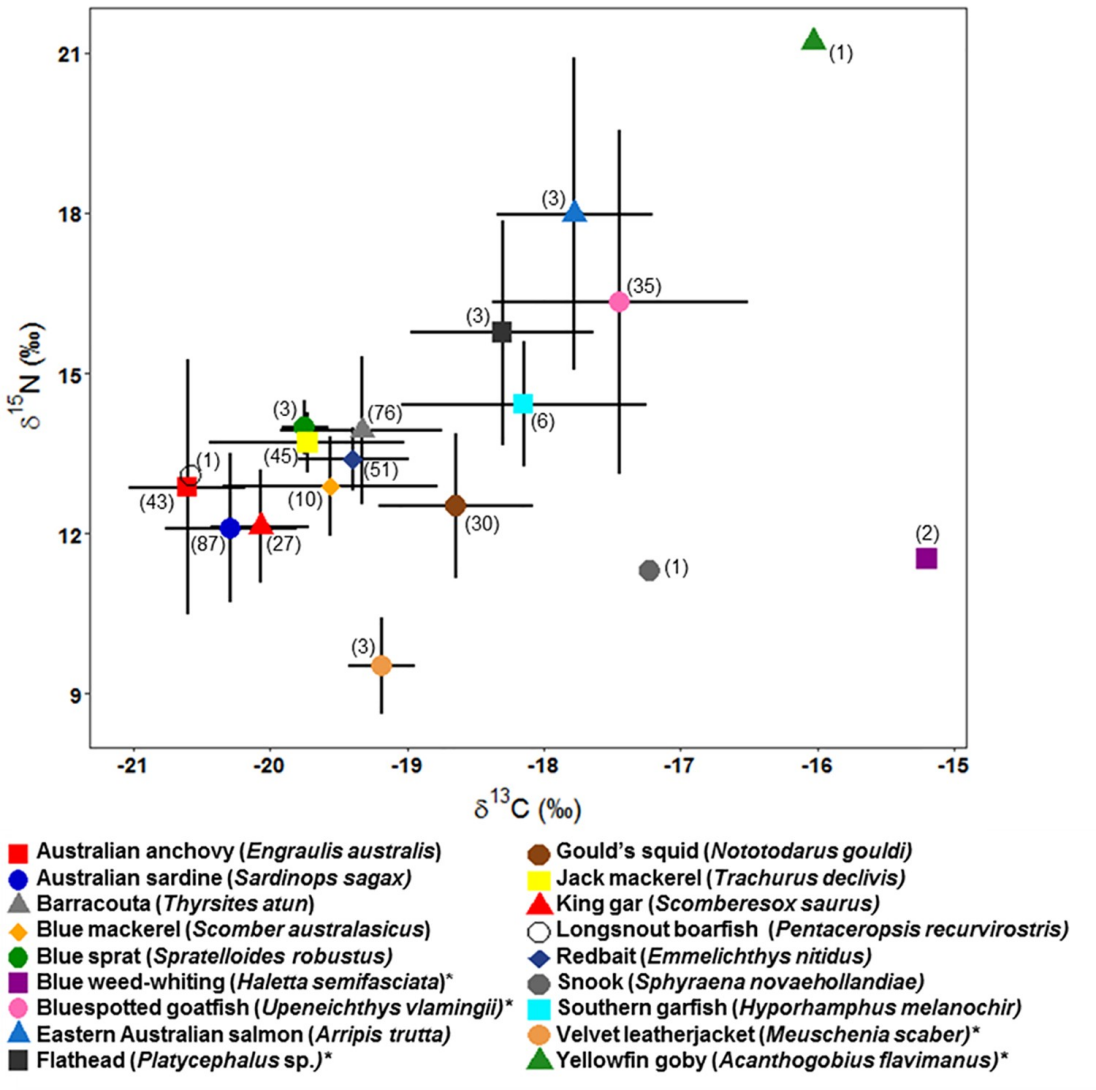
Stable isotope biplot indicating the mean ± SD of *δ*^13^C and *δ*^15^N positions of the 18 prey species collected from Australasian gannet (*Morus serrator*) regurgitates at the Point Danger and Pope’s Eye breeding colonies. Numbers in parentheses represent the total number of prey individuals analysed. Asterisks on species name identify benthic species, all others are considered pelagic.

A wide range of *δ*^13^C and *δ*^15^N values were observed among the species identified (Fig 2). There was a clear distribution in the isotopic space among the prey species collected, from the species with the most depleted values in ^13^C, the pelagic Australian anchovy (*Engraulis australis*, −20.60 ± 0.42 ‰ SD), to the least depleted, the benthic blue weed-whiting (*Haletta semifasciata*, −15.20 ± 2.63 ‰ SD). The *δ*^13^C mean range for all species was 1.73 ± 1.28 ‰ SD. The *δ*^15^N values ranged from the velvet leatherjacket (*Meuschenia scaber*, 9.51 ± 0.89 ‰ SD), a mostly benthic invertebrate feeder, to the yellowfin goby (*Acanthogobius flavimanus*, 21.21 ‰), a crustacean and fish predator. The *δ*^15^N mean range for all species was 4.46 ± 3.88 ‰ SD.

**Fig 3:**
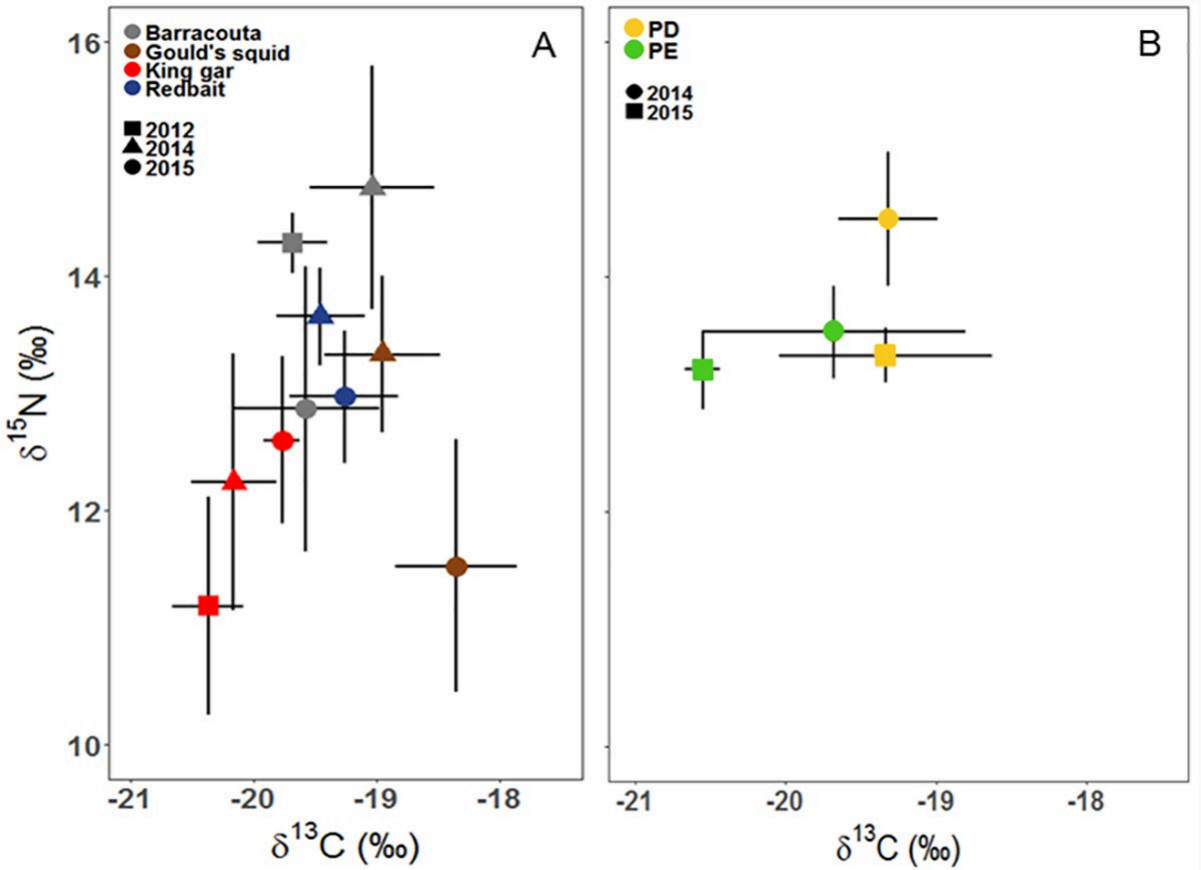
Stable isotope biplot indicating the mean (± SD) of *δ*^13^C and *δ*^15^N values for different prey species. A. Inter-annual comparisons with significant results: barracouta (*Thyrsites atun*), Gould’s squid (*Nototodarus gouldi*) and king gar (*Scomberesox saurus*) in both *δ*^13^C and *δ*^15^N values, redbait (*Emmelichthys nitidus*) in *δ*^15^N values only. B. Inter-annual and geographic (Point Danger = PD and Popes Eye = PE) comparison with significant results: jack mackerel (*Trachurus declivis*) for *δ*^15^N values only.

Eight prey species were collected with sufficient sample sizes for investigating temporal and spatial variability. Mean *δ*^13^C values differed among three prey species, with significant temporal differences. In five cases, there were significant interannual differences. For barracouta (*Thyrsites atun*), 2014 values were higher compared to 2012 and 2015. In contrast, values for *Redbait (Emmelichthys nitidus)*, Gould’s squid (*Nototodarus gouldi)*, and king gar (*Scomberesox sauri)* presented higher values in 2015 than in other years. There were, however, no significant differences in prey *δ*^13^C values between colonies (Table 2, Fig 3).

**Table 2:** ANOVA test results for temporal and spatial differences in δ^13^C prey values (mean ± SD). Significant results of the ANOVA test are shown and *P-*values only provided for non-significant tests. Means with the same superscript denote homogenous subset (*P* > 0.05). Sample sizes indicated in Table 1.

Species	ANOVA test	2012		2013		2014		2015	
		PD (‰)	PE (‰)	PD (‰)	PE (‰)	PD (‰)	PE (‰)	PD (‰)	PE (‰)
Australian anchovy	*P* = 0.25					-20.09 ^a^	-20.36 ± 0.2 ^a^	-20.56 ± 0.4 ^b^	-20.81 ± 0.4 ^b^
Australian sardine	*P* = 0.16					-20.12 ± 0.4	-20.30 ± 0.3	-20.33 ± 0.5	-20.46 ± 0.5
Barracouta	Year, F_2,65_ = 3.90, *P* = 0.025	-19.55 ± 0.1 ^a^	-20.04 ^a^			-19.03 ± 0.5 ^b^	-19.03 ± 0.5 ^b^	-19.54 ± 0.5 ^a^	-19.58 ± 0.6 ^a^
Bluespotted goatfish	*P* = 0.91		-17.59 ± 0.0		-17.16 ± 0.8		-17.64 ± 0.7		-17.33 ± 1.3
Gould’s squid	Year, F_1,23_ = 9.14, *P* = 0.006					-18.94 ± 0.4 ^a^		-18.35 ± 0.4 ^b^	
Jack mackerel	*P* = 0.26					-19.31 ± 0.3	-19.68 ± 0.8	-19.33 ± 0.7	-20.55 ± 0.1
King gar	Year, F_2,24_ = 9.31, *P* < 0.001	-20.36 ± 0.3 ^a^				-20.15 ± 0.3 ^a^		-19.76 ± 0.1 ^b^	
Redbait	Year, F_1,51_ = 5.77, *P* = 0.02					-19.45 ± 0.3		-19.26 ± 0.4	

Breeding colonies: PD = Point Danger, PE = Pope’s Eye.

Mean *δ*^15^N values differed among five prey species, with significant temporal differences in all species. Similar to *δ*^13^C values, 2015 *δ*^15^N values were significantly different from the other years sampled. For king gar, values in 2015 were higher than in 2012 and 2014, while for barracouta, Gould’s squid and redbait (*Emmelichthys nitidus)* 2015 values were lower than other years sampled. Jack mackerel (*Trachurus declivis)* was the only prey species that had significant differences between colonies, as well as an interaction between years and colonies. For this species, however, the difference between the colonies was only detected due to the higher values of PD-2014 (Table 3, Fig 3).

**Table 3:** ANOVA test results for temporal and spatial differences in δ^15^N prey values (mean ± SD). Significant results of the ANOVA test are shown and *P-*values only provided for non-significant tests. Means with the same superscript denote homogenous subset (*P* > 0.05). Sample sizes indicated in Table 1.

Species	ANOVA test	2012		2013		2014		2015	
		PD (‰)	PE (‰)	PD (‰)	PE (‰)	PD (‰)	PE (‰)	PD (‰)	PE (‰)
Australian anchovy	*P* = 0.65					13.73	13.41 ± 1.7	12.31 ± 0.7	13.22 ± 3.8
Australian sardine	*P* = 0.97					12.13 ± 0.5	12.44 ± 1.9	12.20 ± 0.6	11.55 ± 1.8
Barracouta	Year, F_2,65_ = 10.48, *P* < 0.001	14.36 ± 0.2^a^	14.03^a^			14.48 ± 0.5^a^	15.10 ± 1.3^a^	12.69 ± 1.1^b^	12.97 ± 1.2^b^
Bluespotted goatfish	*P* = 0.97		18.10 ± 4.1		16.29 ± 2.5		15.57 ± 3.0		17.10 ± 3.9
Gould’s squid	Year, F_1,23_ = 21.90, *P* < 0.001					13.33 ± 0.6^a^		11.52 ± 1.0^b^	
Jack mackerel	Year, F_1,31_ = 16.07, *P* < 0.001					14.49 ± 0.5^a^	13.52 ± 0.4^b^	13.32 ± 0.2^b^	13.20 ± 0.3^b^
	Colony, F_1,31_ = 27.51, *P* < 0.001								
	Interaction, F_1,31_ = 5.20, *P* = 0.03								
King gar	Year, F_2,24_ = 4.1, *P* = 0.02	11.18 ± 0.9^a^				12.24 ± 1.1^b^		12.59 ± 0.7^b^	
Redbait	Year, F_1,47_ = 23.89, *P* < 0.001					13.65 ± 0.4^a^		12.96 ± 0.5^b^	

Breeding colonies: PD = Point Danger, PE = Pope’s Eye.

**Fig 4:**
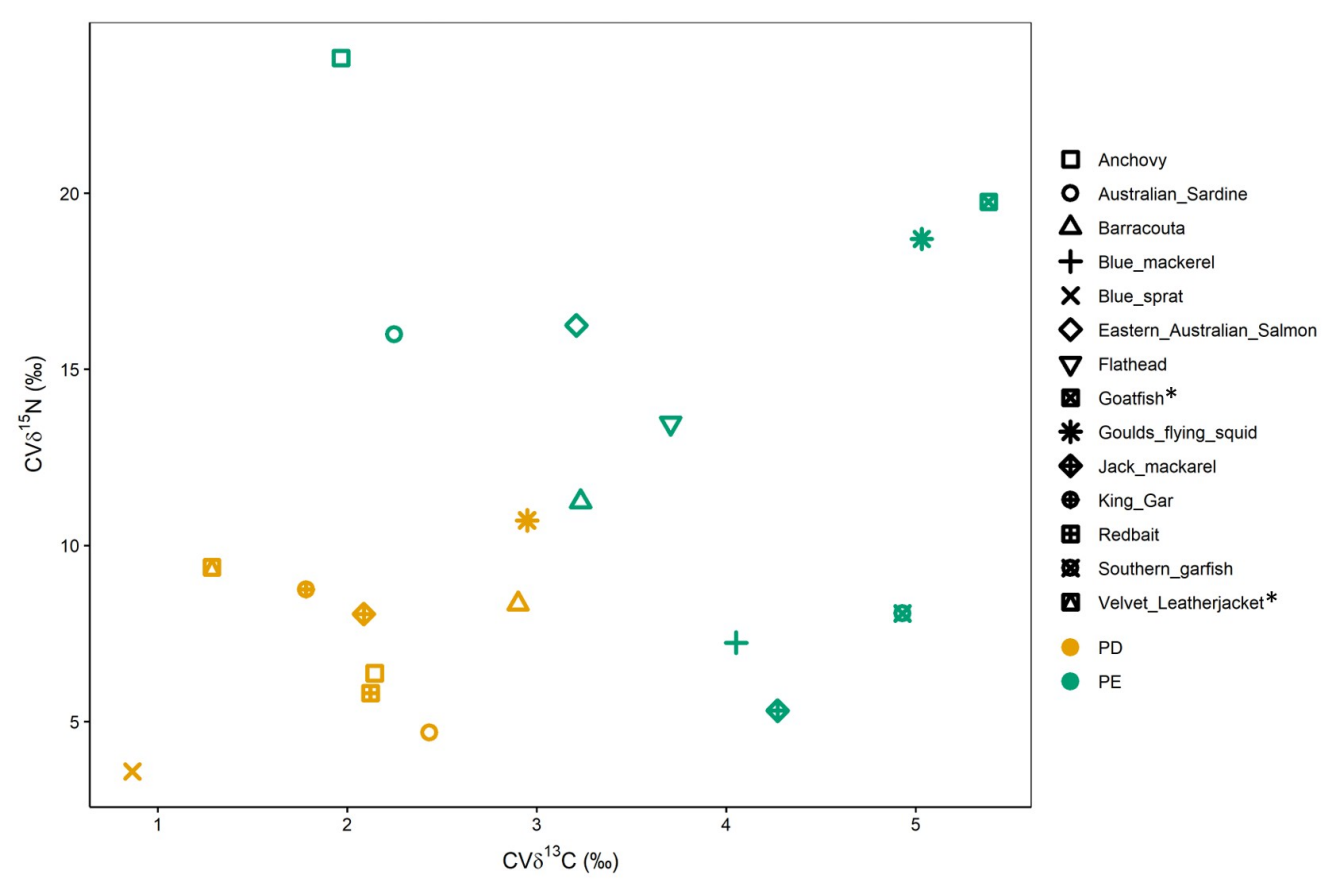
Coefficients of variation in *δ*^13^C and *δ*^15^N values within each colony (Point Danger = PD and Popes Eye = PE) for all prey with ≥ 3 samples collected (n = 14). Asterisks on species name identify benthic species.

The coefficients of variation from prey species within each year and colony ranged from 1% to 8% and from 2% to 29% for *δ*^13^C and *δ*^15^N, respectively. Samples collected at PE, particularly for benthic species, generally presented a higher relative degree of variability in both isotopes than those samples collected at PD (Fig 4). The equality test showed significant results between the species partition (*D’ AD* = 163.78, *P* <0.0001), concluding the species values are significantly more variable in benthic environments.

## Discussion

The findings of the present study indicated significant inter-annual variation in both the *δ*^13^C and *δ*^15^N values of several prey species, suggesting temporal fluctuations in their geographic source or the origin of primary producers supporting prey species. This has significant implications for the interpretation of predator diets from analyses of stable isotopes in tissues. This is especially so as several of the prey species analysed (e.g. barracouta, jack mackerels, redbait and Gould’s squid) are important food items for a range of other predators in the region such as southern blue-fin tuna, Australian fur seals, little penguins and sharks [45, 57–60].

The *δ*^13^C values of lower trophic level prey species can be influenced by temporal and spatial variations in the carbon isotope composition of phytoplankton. Although small, the significant inter-annual differences in *δ*^13^C values observed in the present study for barracouta, Gould’s squid and king gar are consistent with previous studies of fish and squid that have documented similar temporal variation [61, 62]. As there was no evidence that the sampled Australasian gannets changed their foraging areas during the study [32, 40, 63, 64], this suggests the isotopic values of these prey (or their nutrients) do not necessarily reflect the area in which they were consumed. While the observed variation may be due to fluctuations in the major currents influencing the area [65, 66], previous studies [47, 67–69] have shown that barracouta, Gould’s squid and king gar are highly mobile in the south-east Australia region. Therefore, differences present in the isotopic values could also be an indication of inter-annual movement of prey within and from outside Bass Strait.

The *δ*^15^N values of species reflect their trophic position [10, 47, 67–69]. In the present study, five species displayed significant inter-annual differences in their *δ*^15^N values: king gar [47]; jack mackerel and redbait [70]; barracouta [70, 71]; and Gould’s squid [72] (diet information presented in Table 1). The observed temporal variation in the *δ*^15^N values of these species could reflect changes in their diet composition or the oceanic source of their nutrients [62]. Indeed, while jack mackerel and redbait are not considered migratory [73, 74], their abundance has been closely linked to the availability of Australian krill (*Nyctiphanes australis*) [75]. The abundance of this euphausiid is influenced by regional oceanography on both seasonal and inter-annual scales [76]. Variability in biogeochemical processes (e.g. N_2_ fixation processes in the surface ocean, terrestrial runoff or atmospheric precipitation [27, 28] may potentially result in *δ*^15^N variation. In addition, individuals occupying the same trophic niche may predate each other. Indeed, barracouta and Gould’s squid, two species sampled in the present study, have been reported to prey on each other [72] which may result in variation of *δ*^15^N values. This variation highlights the potential problem in inferring changes to the diet of higher predators from tissue stable isotope values without concurrent information on prey base values [21, 22].

Interestingly, most of the significant inter-annual variation in both *δ*^13^C and *δ*^15^N values occurred in 2015, which coincided with a strong El Niño-Southern Oscillation (ENSO) event with widespread below-average rainfall and higher (≥ 2° C) SST across south-eastern Australia [77]. These extreme conditions could potentially alter the range and spawning areas of the sampled species [78–80], their growth rates [81] and their migration routes [82]. Additionally, the physical changes associated with ENSO events have been shown to influence marine primary production with subsequent impacts on secondary and tertiary consumers through changes in prey availability [83, 84]. However, the significant differences found in some species could also be related to differences in sample size between years (2012 and 2013 had smaller sample sizes than 2014 and 2015).

In contrast, five prey species did not display significant temporal or spatial variability in their *δ*^13^C values. Except for the blue-spotted goatfish, an inhabitant of the sandy sea floor of Port Phillip Bay [85], the species (Australian anchovy and sardine, jack mackerel and redbait) are pelagic and abundant in inshore and shelf waters of eastern Australia during summer and autumn [47, 60]. Similarly, the Australian anchovy and sardine, two planktivorous species, and the blue-spotted goatfish, a consumer of benthic invertebrates, showed no significant temporal or spatial variation in the *δ*^15^N values. The findings suggest that these prey species did not experience a significant diet change during the study.

There was a substantial range in the coefficients of variation of isotope values within sampling periods and sites. Samples collected at PE, most notably those of benthic species, generally displayed higher relative variation in isotope values that those collected at PD. Gannets at the PE breeding colony feed in both Port Phillip Bay, a shallow water body adjacent to the city of Melbourne with constant freshwater input from rainfall, rivers, creeks and drains [86], and in the northern Bass Strait, an area influenced by multiple currents over short time scales [31, 32]. The results of the present study suggest these features may lead to fine-scale spatial isoscape variation for the region’s fish prey species [29, 30]. In contrast, gannets from PD forage in the continental shelf area of western Bass Strait with relatively uniform oceanographic influences during the breeding season [69, 87]. Such factors should be considered when inferring diet composition from tissue stable isotope values in predators.

Stable isotope analysis is a powerful tool for qualitatively and quantitatively assessing animal diets [3, 5]. It has been shown that the isotopic differences seen in a predator diet across time may be the result of a change in diet composition (either in different prey or prey ratios) [15, 16], a change in the feeding location or the combination of both [13, 14]. As has been shown elsewhere in previous studies [22, 62, 88], the present study has demonstrated temporal variation in the isotopic values of some common marine prey species within south-eastern Australia. While the differences among the annual means of the prey isotope values were small, if these prey species constitute a large proportion of a predator’s diet, it could substantially affect interpretations of predator isotope values. Similarly, geographic variation in prey isotopic values can affect interpretations of predator diets if they occur within their foraging range [30]. For example, the predictive power of isotopic mixing models can be compromised if the isotopic values of specific prey varies substantially over temporal scales of a study or spatial scales over which the predators move [12]. Therefore, it is advisable to use isotopic data from marine preys collected within the same location and time period of the predators under study [12, 21]. In conclusion, it is important to consider the potential significant differences in prey species isotopic values within the assemblages that predators consume to interpret correctly their isotopic variability and better understand the influence of the environment on their diets.

## Supporting information

S1 Table Means (± SE) of the standard length and body mass for prey species by year and colony (where whole specimens or otoliths could be measured). Sample sizes may vary from those used for statistical analysis.
(DOCX)
