# Supplementary material for: Temporal and spatial isotopic variability of marine prey species in south-eastern Australia: Potential implications for predator diet studies
Source: PLoS One. 2021 Nov 30;16(11):e0259961. doi: 10.1371/journal.pone.0259961 (PMC8631622; doi:10.1371/journal.pone.0259961)
Supplement: S1 Table — Sample sizes may vary from those used for statistical analysis. (DOCX) [file pone.0259961.s001.docx]

The following supplement accompanies the article

**Temporal and spatial isotopic variability of marine prey species in south-eastern**

**Australia: potential implications for predator diet studies**

Marlenne A. Rodríguez-Malagón^1^, Cassie N. Speakman^1^, Grace J. Sutton^1^, Lauren P. Angel^1^ and John P.Y. Arnould*^1^

^1^Deakin University, Geelong, Victoria 3220, Australia

School of Life and Environmental Sciences (Burwood Campus)

*Corresponding author: john.arnould@deakin.edu.au

| Species | Year | Colony | n | Standard length (mm) | Body mass (g) |
| --- | --- | --- | --- | --- | --- |
| Australian anchovy | 2014 | PD | 1 | 87.2 | 6.2 |
| Australian anchovy | 2014 | PE | 14 | 96.6 ± 2.5 | 9.1 ± 0.8 |
| Australian anchovy | 2015 | PD | 24 | 99.3 ± 1.8 | 10.8 ± 0.7 |
| Australian anchovy | 2015 | PE | 23 | 98.4 ± 2.1 | 9.5 ± 0.7 |
| Australian sardine | 2013 | PE | 3 | 116.7 ± 21.2 | 16.0 ± 6.5 |
| Australian sardine | 2014 | PD | 162 | 101.7 ± 3.2 | 17.3 ± 1.6 |
| Australian sardine | 2014 | PE | 42 | 123.3 ± 3.7 | 23.0 ± 2.2 |
| Australian sardine | 2015 | PD | 20 | 147.1 ± 8.9 | 39.6 ± 4.5 |
| Australian sardine | 2015 | PE | 52 | 123.1 ± 5.5 | 25.5 ± 2.8 |
| Barracouta | 2014 | PD | 5 | 199.9 ± 64.6 | 66.8 ± 57.1 |

Table S1: Means (± SE) of the standard length and body mass for prey species by year and colony (where whole specimens or otoliths could be measured). Sample sizes may vary from those used for statistical analysis

Barracouta 2014 PE 9 321.6 ± 51.7 299.8 ± 73.5

Barracouta 2015 PD 16 187.7 ± 24.8 52.3 ± 24.8

Barracouta 2015 PE 17 236.1 ± 19.1 73.2 ± 29.9

Blue mackerel 2014 PE 2 291.5 ± 25.5 307.7 ± 100.4

Blue mackerel 2015 PE 1 310.0 461

Bluespotted goatfish 2013 PE 3 181.6 ± 18.3 118.4 ± 32.8

Bluespotted goatfish 2014 PE 1 172.0 121.4

Bluespotted goatfish 2015 PE 4 141.5 ± 6.4 56.3 ± 3.7

Flathead 2013 PE 1 211.0 81.8

Gould's squid 2012 PD 1 425.0 305.0

Gould's squid 2014 PD 5 117.2 ± 20.0 44.2 ± 18.2

Gould's squid 2014 PE 2 128.4 ± 11.5 103.9 ± 64.0

Gould's squid 2015 PD 11 120.4 ± 9.0 10.7 ± 1.5

Jack mackerel 2012 PE 7 225.6 ± 6.7 131.5 ± 10.9

Jack mackerel 2014 PD 4 259.7 ± 2.4 183.7 ± 23.2

Jack mackerel 2014 PE 10 241.4 ± 7.9 173.0 ± 17.6

Jack mackerel 2015 PD 2 256.7 ± 9.7 195.6 ± 15.6

Jack mackerel 2015 PE 3 244.6 ± 4.8 161.18 ± 8.3

King gar 2014 PD 2 200.0 ± 7.0 21.3 ± 1.9

Redbait 2014 PD 9 199.8 ± 2.1 94.6 ± 2.9

Redbait 2015 PD 16 137.4 ± 3.9 36.9 ± 3.3

Southern garfish 2014 PE 3 214.8 ± 26.9 26.4 ± 12.2

Southern garfish 2015 PE 1 300.0 89.0

Velvet leatherjacket 2014 PD 1 162.0 83.2

Yellowfin goby 2015 PE 1 180.0 38.0

13
